# Changes in circulating filarial antigen status in previously positive individuals: Lessons for treatment monitoring and pre-transmission assessment surveys

**DOI:** 10.1371/journal.pntd.0012802

**Published:** 2025-02-04

**Authors:** Derrick Adu Mensah, Vera Serwaa Opoku, John Boateng, John Opoku, Jubin Osei-Mensah, Charles Gyasi, Prince Obeng, Monica Ahiadorme, Prince Dennis Atisu, Emmanuel Donawobuge Kutu, Inge Kroidl, Ute Klarmann-Schulz, Achim Hoerauf, Linda Batsa Debrah, Alexander Yaw Debrah

**Affiliations:** 1 Department of Clinical Microbiology, School of Medicine and Dentistry, Kwame Nkrumah University of Science and Technology, Kumasi, Ghana; 2 Kumasi Centre for Collaborative Research in Tropical Medicine, Kwame Nkrumah University of Science and Technology, Kumasi, Ghana; 3 Department of Pathobiology, School of Veterinary Medicine, Kwame Nkrumah University of Science and Technology, Kumasi, Ghana; 4 Division of Infectious Diseases and Tropical Medicine, LMU University Hospital, LMU Munich, Munich, Germany; 5 German Center for Infection Research (DZIF), partner site Munich, Munich, Germany; 6 Institute for Medical Microbiology, Immunology and Parasitology, University Hospital Bonn, Bonn, Germany; 7 German Center for Infection Research (DZIF), partner site Bonn-Cologne, Bonn, Germany; 8 German-West African Center for Global Health and Pandemic Prevention (G-WAC), partner site Kumasi, Kumasi, Ghana; 9 Department of Medical Diagnostics, Faculty of Allied Health Sciences, Kwame Nkrumah University of Science and Technology, Kumasi, Ghana; University of Sussex, UNITED KINGDOM OF GREAT BRITAIN AND NORTHERN IRELAND

## Abstract

**Background:**

The Global Programme to Eliminate Lymphatic Filariasis has made significant gains through mass drug administration (MDA) of Ivermectin/Albendazole. Periodic evaluation of the MDA programme in lymphatic filariasis elimination is particularly useful in determining end points for stopping the programme. This is a follow-up study that sought to examine the effects of additional time and MDA intake on antigenemia seroreversion in persons who had previously tested positive for LF using the Filarial Test Strip (FTS) and the TropBio ELISA over a period of 1–5 years.

**Methodology/principal findings:**

A total of 542 individuals, from the Kassena Nankana East Municipal (N = 340) and Nabdam districts (N = 202) in the Upper East Region of Ghana, who had previously tested either positive (N = 446) or negative (N = 96) for FTS-CFA, participated in the study. Two follow-up visits were conducted; 1–4 years (follow-up-1) and 2–5 years (follow-up-2) after the baseline visit. Of the 446 FTS-CFA positives, 175 (39.2%) did not receive additional MDA (ivermectin/Albendazole) after the baseline visit. Overall, from the two follow-up visits, 159/175 (90.9%) FTS-CFA+ participants who did not receive any additional Ivermectin/Albendazole and 226 out of the 271 (83.4%) with additional MDA treatment became CFA negative. A total of 120 randomly selected baseline FTS-CFA+ samples were tested with Og4C3 TropBio ELISA and only 44/120 (36.7%) were found positive. Of these 44 participants, 12 (27.3%) completely became CFA negative and an additional 18 (40.9%) had reduced antigen levels during the follow-up. Likewise, all three previously/baseline microfilariae positive persons had become amicrofilaremic.

**Conclusions/significance:**

In the present work, it has been shown that >90% of the previous CFA positive individuals seroreverted in 1 to 5 years post-baseline without additional MDA. The FTS is a more sensitive diagnostic tool that plausibly detects residual CFA in blood. The impact and influence of time as compared to additional ivermectin/Albendazole intake, on CFA seroreversion in this study was significant (p < 0.001).

## Author summary

When GPELF started, between 5 to 6 effective rounds of MDA covering at least 65% of the endemic population was thought to be sufficient to halt LF disease transmission. The pre-Transmission Assessment Survey (pre-TAS), was the standardized method of LF prevalence testing adopted by the WHO. The Alere Filarial Test Strip (FTS) used in LF prevalence testing is known to detect low concentrations of CFA and therefore may likely detect persisting residual CFA following the death of the adult worms. Studies have indicated an increased risk of pre-TAS failure when the FTS Alere kit is used to assess treatment impact on antigenemia levels among adults in sentinel sites with <1% MF prevalence. This was a 1 to 5-year follow-up study which assessed the impact of time and Ivermectin/Albendazole intake on LF antigenemia seroreversion/seroconversion in individuals who had previously tested either positive or negative to the FTS. The results of this study indicated that a large percentage (91%) of the previously CFA positive participants had become CFA negative without intake of additional ivermectin/Albendazole. In addition, there was no CFA seroconversion in all the previously CFA negative participants. Time (years of follow-up) had significant impact and influence on FTS antigenemia seroreversion.

## Introduction

Lymphatic filariasis (LF), caused by the parasitic nematodes *Wuchereria bancrofti* or *Brugia* species, is a leading cause of chronic morbidity and disability [1]. Globally, about 882.5 million people in 44 countries are at risk of LF infection, with 51 million currently infected and approximately 36 million people suffer severe disabling complications such as hydrocele and lymphedema, which may progress to elephantiasis [2]. The World Health Organization (WHO) through the Global Programme to Eliminate Lymphatic Filariasis (GPELF) has set two main goals for global elimination of LF by 2030 [2,3]. The first goal is to interrupt the disease transmission through the usage of mass drug administration (MDA) programmes in endemic areas. The second goal is to ameliorate and manage the morbidities associated with chronic cases of LF to improve quality of life [3]. Since 2000, the GPELF has made impressive gains, with a total of 7.7 billion treatments delivered to over 890 million people residing in endemic areas all over the world [2]. Within the elimination programme, at least 65% of the total population at risk should be reached through MDA, followed by effective monitoring and surveillance to ensure that programmatic gains relating to disease interruption are sustained in the long-term [3,4].

In Ghana, the Filariasis Elimination Programme, which was founded in 2000 in the then 98 mapped endemic districts, has made significant strides toward LF elimination [5,6]. At the outset, the baseline microfilariae (MF) prevalence was 8.7% on average; ranging between 0 to 45.7% in the various mapped sentinel areas [5]. Through this programme, there has been a rapid decline in MF prevalence after the first few years of MDA, followed by a steady decline in the years thereafter [5,6]. Recorded MF prevalence differed highly after 6 to 7 rounds of MDA in places where the MF prevalence often exceeded 1% or even 5%. However, by the end of 2016 and an average of 11 rounds of treatment, 81 out of the then 98 endemic districts in Ghana had met the WHO criteria for stopping MDA [5]. As at the beginning of 2017, MF prevalence in the remaining 17 districts was more than 1%, and therefore these had been termed hotspot districts [5]. However, according to the Ghana Neglected Tropical Diseases Programme (NTDP), only seven of such LF hotspots currently remain in this end-game scenario. Communities in the Kassena Nankana East Municipal (KNEM) and Nabdam districts, which started MDA in 2000 and 2005, respectively, are two of such LF hotspots in the Upper East Region of Ghana [5].

Using the BinaxNOW Filariasis immunochromatographic Card Test (ICT) to determine antigenemia, baseline antigen prevalence was found to have greatly reduced from 45.4% in 2000 to 6.6% after seven years of MDA initiation in KNEM. Also, using microscopy to detect MF in KNEM, MF prevalence was found to have remarkably reduced from 29.6% at baseline in 2000 to and 4.0% in 2007 [5]. However, due to challenges with the diagnostic and operational characteristics, the usage of the BinaxNOW ICT in LF antigenemia monitoring was discontinued [7]. Subsequently, the Alere Filariasis Test Strip (FTS) was introduced, with increased sensitivity and improved operational characteristics over the BinaxNOW ICT, thus satisfying the Neglected Tropical Diseases Strategic and Technical Advisory Group’s Monitoring and Evaluation Sub-group on Disease Specific Indicators (DSI) [7]. There was an agreement among members of the sub-group that the increased sensitivity of the FTS in comparison to ICT was acceptable for the purposes of the GPELF, and that the antigenemia critical cut-off for passing pre-Transmission Assessment Surveys (pre-TAS) of circulating filarial antigen (CFA) less than 2%, remains unchanged [7].

Findings from a recent study conducted by our group between June 2018 to May 2021 in the KNEM and Nabdam districts suggested that the FTS detected significantly more filarial antigen positives in comparison to TropBio Og4C3 ELISA [8], similar to the findings from another study by Chesnais *et al* [9]. In the former study [8], both the FTS and TropBio Og4C3 ELISA antigen prevalences (17% and 3.8%, respectively) did not meet the 2% CFA threshold, and thus suggestive of LF transmission with FTS-CFA prevalence also being significantly higher than MF prevalence (0.14%) [8]. The findings from the above-referenced study indicated ongoing transmission in these two sentinel sites based on antigen prevalence by FTS and TropBio Og4C3 ELISA tests, but not by MF microscopy. MF prevalence on the contrary suggested a halt in LF transmission in both districts [8]. The FTS is known to detect low concentrations of CFA [7,10], and may also likely detect persisting residual CFA following the death of the adult worms [11,12]. However, given that residual antigens may decline sharply after a few years, we hypothesize that antigen prevalence would be significantly lower in the years that follow our baseline screening, and should consequently be lower than what was reported in our most recent study, which was also conducted in these two hotspot areas [8].

Only few longitudinal studies on the FTS’ propensity in detecting “persistent residual circulating filarial antigen” have been conducted. Therefore, in this article, we report results of 1–5 years follow-up study, conducted in 542 individuals who previously had tested either positive or negative to FTS, to assess the impact of time and ivermectin/Albendazole intake (IVM/ALB) on antigenemia seroreversion (antigen positive to negative) or seroconversion (antigen-negative to positive) in this cohort of individuals. The objective was to confirm whether these individuals were truly antigen positive as earlier reported [8], or their positivity was as a result of residual antigen materials from dead adult worms circulating in the blood, as has been suggested by others [11,12]. Furthermore, with the current 2030 LF elimination roadmap target drawing closer, the need for refinement of strategies for re-assessment of transmission, as well as the period needed to undertake such transmission re-assessment studies is necessary and needs to be clearly answered.

## Methods

### Ethics statement

Approval for this study was obtained from the Committee on Human Research Publications and Ethics (CHRPE) of the School of Medicine and Dentistry at the Kwame Nkrumah University of Science and Technology (KNUST), Kumasi, Ghana. Additional approvals were obtained from the Upper East Regional, KNEM and Nabdam District Health Directorates, as well as permission sought from the community opinion leaders and elders. Study purpose and procedures were clearly explained to the participants in their local languages. A written informed consent was obtained from all participants either by signing or thumbprinting an ethically approved Informed Consent Form which was administered in English, “Twi”, “Nabt”, “Kassim” or “Nankani”, depending on the participant’s choice of language. This study was carried out as part of other larger funded projects, namely: “Tackling the Obstacles to Fight Filariasis and Podoconiosis” (TAKeOFF), the First Stage Genome Wide Association study of Lymphatic Filariasis pathology (GWAS) and the Alternative treatment Strategies using anti-wolbachial drugs to accelerate elimination of Lymphatic Filariasis and Onchocerciasis (ASTAWOL). The ethical approval registration numbers for the above studies were CHRPE/AP/525/17, CHRPE/AP/366/17 and CHRPE/AP/337/20 respectively.

### Study setting and population, MDA with Ivermectin and Albendazole profile

The research was carried out in the Kassena-Nankana East Municipal (KNEM) and Nabdam districts, in the Upper East Region of Ghana. The study areas have previously been described [8]. The study was conducted in 28 LF-endemic communities in three sub-districts of KNEM (Pungu, Navrongo-East and the Manyoro sub-districts) and 33 LF-endemic communities in five sub-districts (Nagodi, Pelungu, Sakote, Zanlerigu and Kongo Pitanga) in the Nabdam district. The KNEM and Nabdam districts started MDA in 2000 and 2005, respectively [5,8]. MDA with ivermectin (IVM) and albendazole (ALB) has been ongoing since our initial screening assessments in 2018 and 2019/2020 for KNEM and Nabdam, respectively, except for the year 2020 due to the COVID-19 pandemic that resulted in a postponement of the MDA programme [13]. The number of IVM and ALB rounds distributed to communities since the initial screening assessments were four and three for KNEM and Nabdam, respectively. All the study communities had undertaken more than 15 MDA rounds, with participants having an average MDA intake of 9 rounds—the most recent being in June/July 2022. In our previous assessment of the burden of the LF disease and infection in KNEM and Nabdam, we reported an antigenemia (FTS-CFA) and MF prevalence of 19.6% and 0.7% for KNEM and 12.8% and 0.5% for Nabdam, respectively [8]. Participants identified to be FTS-CFA positive were followed-up in this study to assess the impact of time and additional ivermectin/Albendazole intake on the clearance or persistence of *W. bancrofti* CFA. In addition, a cohort of FTS-CFA negative individuals were followed-up to evaluate potential new infections.

### Study design

This was a longitudinal study conducted from June 2018 to July 2023 using a purposive community sampling technique. Individuals living in the endemic communities were first screened for LF antigen between June 2018 to May 2021, with both antigen positive and negative individuals followed-up twice; first follow-up– June to November 2022 (1–4 years) and a second follow-up– July 2023 (2–5 years) for a possible change in antigen status—after the initial screening and additional MDA distribution by the National NTD programme. The second follow-up was purposely done to assess a possible change in antigenemia status of participants who still remained FTS-CFA+ during the first follow-up. Dissemination of information to participants for the follow-up assessments were given through the community health volunteers (CHVs) and informed consenting was carried out as previously described in our initial study [8]. Only individuals able to give consent were included in the study. For a prevalence of 17.0% (previous study) [8], 0.05 margin of error and a confidence interval of 95, a minimum of 216 participants each for FTS-positives (testing for seroreversion) and FTS-negatives (testing for seroconversion) groups were required for the follow-up study to ensure statistical power. After the study explanations, the participants were invited to ask questions and these were answered by the research team satisfactorily.

### Recruitment of study participants and data collection

A total of 542 participants comprising 340 from KNEM and 202 from the Nabdam district, aged between 14 to 59 years were followed-up in this study. These participants (N = 542) were drawn from a former study [8], which tested for the prevalence of lymphatic filariasis infection after 15 years of MDA in two endemic foci in the Upper East Region of Ghana. Out of 1264 CFA positives recorded in the previous study [8], 446 consented participants were randomly selected from some selected communities in the previous study―and out of the 6189 CFA negatives, 96 were recruited. These were those who voluntarily consented to participate in the current follow-up study after having their CFA status declared to them privately at the end of the previous study. Thus, all reported prevalences in this current study are prevalence estimates based on the voluntary participation in the study and not an epidemiological pre-defined sample size. In this referenced study [8], both the FTS-CFA (12.8%–19.6%) and TropBio Og4C3 ELISA (3.8%) antigen prevalences did not meet the < 2% CFA criterion which is suggestive of LF elimination—with FTS-CFA prevalence also being significantly higher than MF prevalence (0.14%) [8]. Also, 120 participants out of the 446 FTS-CFA baseline positives were randomly selected for Og4C3 CFA TropBio ELISA testing. These participants were selected based on availability of baseline ELISA results. Participants were asked for the number of additional MDA taken since the initial screening in 2018/2021, and this was orally declared. However, to prevent recall bias, responses from participants were cross-checked with that of the CHVs’ household MDA records, if available, as was done in the initial study [8].

### Laboratory investigations

All methods regarding the re-evaluation of antigenemia and MF status of the participants for LF infection was done as previously described [8]. In summary, finger prick capillary blood was used for the FTS antigen test and read within 10 minutes independently by two investigators following the manufacturer’s instructions. For MF assessment by microscopy and TropBio Og4C3 CFA ELISA (TropBio; Cellabs, Dale Street Brookvale, NSW 2100 Australia; LF2.3; KF1/KF2), 5ml of venous blood in EDTA was taken by trained phlebotomists from FTS positive participants between the hours of 10:00 pm – 12:00 am, due to the nocturnal periodicity of *W. bancrofti* MF [8]. Methods used for MF assessment and quantification have previously been described [8]. Briefly, about 2mL of the venous whole blood night samples were collected into EDTA tubes. Two hundred microliters (200 µL) of the blood sample were diluted in 800µl of 3% acetic acid and transferred into a Sedgewick counting chamber. A confirmatory test was done by filtering 1 mL of whole blood mixed with 8 mL of distilled water through a 5 mm Whatmann Nucleopore filter and then fixed with methanol on a microscope slide. The Filter is air dried, stained with 5% Giemsa and observed under the X10 objective lens of a light microscope. The number of MF counted were recorded as MF/mL.

For the TropBio ELISA, 3.5ml of the blood was centrifuged and the plasma stored at −80 °C for later testing of Og4C3 CFA at the Kumasi Centre for Collaborative Research in Tropical Medicine (KCCR), KNUST, Kumasi. Samples were processed as previously described and analyzed using SpectraMAX Plus 190 at a dual wavelength of 450/520 nm [8]. Results interpretation was done according to the manufacturer’s updated recommendation (August, 2021) for areas which have had many rounds of MDA and has resulted in low LF prevalence (TropBio; Cellabs, Dale Street Brookvale, NSW 2100 Australia; LF2.3; KF1/KF2), which is as follows: the optical density (OD) of standard 2 was used as the cut-off if within +/− 10% of 0.35 or alternatively, a default value of 0.35 was used if standard 2 OD fell outside the range and interpreted as—Negative = any OD below the set cut-off; Low Positive = any OD from the cut-off but below 0.8; Medium Positive = OD from 0.8–1.0 and High Positive = OD *>* 1.0 [8]. FTS-CFA negative participants were not assessed for Og4C3 TropBIO ELISA as this has been seen from our previous studies to carry no active infection.

### Data curation and statistical analyses plan

Data were captured on paper case report forms (CRFs) and later entered into Microsoft Office Excel 2019. These were cross-checked for consistency and accuracy and later cleaned before statistical analyses were done. Statistical analyses were conducted using the IBM SPSS version 26 and GraphPad Prism version 9 for trend analysis and graphing. Descriptive statistics were employed for age, gender, years of follow-up and ivermectin/Albendazole intake. Frequencies of antigenemia and microfilaremia using FTS-CFA, TropBio Og4C3 CFA, and MF were expressed as percentages. The Fisher’s Exact test was used to compare the proportions of various independent categorical variables such as years of follow-up since initial screening and additional ivermectin/Albendazole (MDA) intake for FTS-CFA or TropBio Og4C3 CFA and the McNemar/McNemar-Bowker tests for related categorical variables for FTS-CFA and TropBio Og4C3 CFA. Trend analyses was used in predicting the influence of years of follow-up and additional ivermectin/albendazole intake since initial assessment on FTS-CFA and TropBio Og4C3 CFA seroreversions were conducted using the Chi-Square test for trend or the Cochran-Armitage trend test. The Wilcoxon signed rank test for non-parametric data was used to compare the change in Og4C3 antigen units over time. Spearman’s correlation coefficient was used to assess the relationship of the follow-up antigen levels against the baseline antigen levels, the years of follow-up and additional IVM intake. Detection of statistically significant variation or difference in parameters were considered at an alpha level of 5% and confidence interval of 95% (p < 0.05).

## Results

### Characteristics and general description of participants followed-up

**Fig 1:**
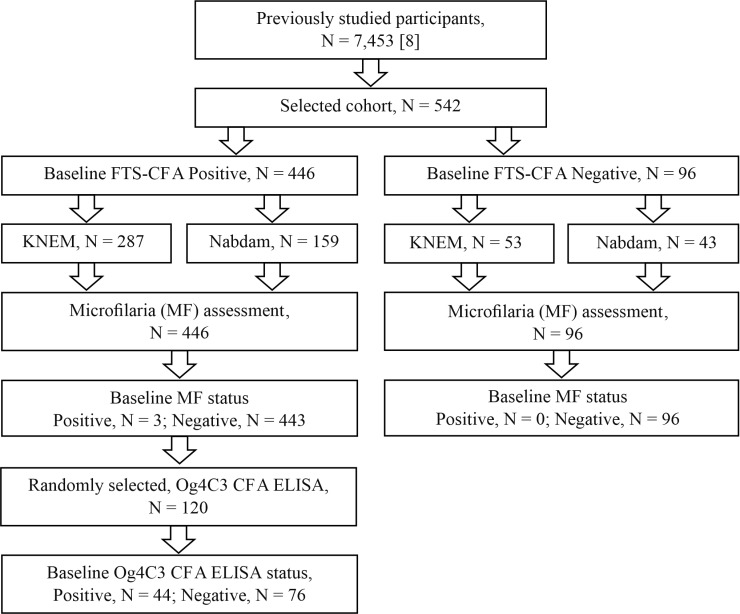
Selection of baseline study cohort.

A cohort of 542 study participants comprising 446 antigen-positive and 96 antigen-negative individuals were drawn from two districts, KNEM and Nabdam and followed-up twice to assess the impact of time and MDA intake on antigenemia seroreversion (antigen positive to negative) or seroconversion (antigen-negative to positive) [Fig 1]. Also, 120 FTS-CFA baseline positive participants (out of the 446 antigen positives) comprising 66 (55.0%) males and 54 (45.0%) females were randomly selected for Og4C3 TropBio testing.

Follow-up time varied for different groups of participants ranging from 1–5 years depending on the initial date of assessment (baseline) [Table 1]. Most of the participants were followed-up within a period of 3 years. Females constituted 56.3% of the cohort of participants and the majority of them (35.1%) were between the ages of 41–50 years (Table 1). In all, 11 out of the 60 communities (18.3%) assessed in KNEM and Nabdam had participants who had never taken part in MDA. In total, 27 (5.0%) of the participants followed-up had never taken MDA (Tables 1 and A in S1 Table). The maximum MDA rounds received were 20. It is worth mentioning that only 3 individuals (0.9%) had received the regular MDA for 20 times in KNEM and 1 individual (0.5%) for 13 times in Nabdam. Most participants, 269 (49.6%) had received 5–10 and 215 (39.7) rounds of MDA with only 31 (5.7%) receiving more than 10 rounds (Tables 1 and B in S1 Table).

**Table 1:** Baseline demographic characteristics of the cohort of participants followed-up.

Variable	Category	First Follow-up N (%)	Variable	Category	Second Follow-up N (%)
**Gender**	Male	237 (43.7)	**Gender**	Male	74 (49.3)
	Female	305 (56.3)		Female	76 (50.7)
**Age groups (years)**	14–30	112 (20.7)	**Age groups (years)**	14–30	25 (16.7)
	31–40	163 (30.1)		31–40	58 (38.7)
	41–50	190 (35.1)		41–50	48 (32.0)
	≥ 51	77 (14.2)		≥ 51	19 (12.7)
**IVM rounds (follow-up)**	0 round	27 (5.0)	**IVM rounds (follow-up)**	0 round	4 (2.7)
	1–4 rounds	215 (39.7)		1–4 rounds	40 (26.7)
	5–10 rounds	269 (49.6)		5–10 rounds	100 (66.7)
	≥11 rounds	31 (5.7)		≥11 rounds	6 (4.0)
**Years of Follow-up**	1 (1.5–1.8)	76 (14.0)	**Years of Follow-up**	2 (2.6–2.9)	27 (18.0)
	2 (2.3–2.9)	126 (23.2)		3 (3.5–3.6)	91 (60.7)
	3 (3.0–3.9)	290 (53.5)		4 (4.0–4.8)	30 (20.0)
	4 (4.0–4.3)	50 (9.2)		5 (5.0)	2 (1.3)
	**Total**	**542 (100.0)**		**Total**	**150 (100.0)**

### Assessment of the incidence of microfilariae infection by microscopy in participants

All FTS-CFA positive participants followed-up were assessed for active MF infection over the first and second follow-up periods. Interestingly, none of the participants (including those that were MF positive at the initial screening) harboured MF in the blood 2–5 years after the initial screening assessment. Hence, the incidence of *W. bancrofti* MF infection in this selected group of participants was 0.0%. Though it might be bias, FTS-CFA negative participants were not assessed as this has been seen from our previous studies to carry no active infection [8].

### Antigenemia seroconversion/seroreversion in participants followed-up – FTS and Og4C3 CFA antigens

All participants followed up were either FTS antigen-negative or antigen positive at the initial screening. For the assessment of seroconversion in previously tested antigen-negative individuals (N = 96), none (0%) of the participants had become antigen positive during the second and final follow-up (Table 2). Irrespective of the follow-up period (whether after 1 or 4 years), 364/446 (81.6%) of all participants who were initially FTS-CFA positive seroreverted to become FTS-CFA negative during the first follow-up. Also, 27.3% (21/77) of the remaining FTS-CFA positive participants had also seroreverted during the second follow-up (Table 5). Thus overall, a total of 385 (86.3%) participants out of the 446 FTS-CFA positives [n = 364 during the first follow-up; n= 21 during the second follow-up] seroreverted throughout the entire study period. Majority of the participants who had seroreverted were from the KNEM.

**Table 2:** Seroconversion/-reversion prevalence in participants followed up.

	Baseline, N (%)	First Follow-up, N (%)	Second Follow-up, N (%)
**FTS (CFA)**			
**Baseline FTS Negative**			
Negative	96 (100.0)	95 (99.0)	44 (100.0)
Positive/ (Seroconversion)	0 (0.0)	1 (1.0)^*^	0 (0.0)
Total (A)	96 (100.0)	96 (100.0)	44 (100.0)
**Baseline FTS Positive**			
Positive	446 (100.0)	82 (18.4)	56 (52.8)
Negative/ (Seroreversion)^**^	0 (0.0)	364 (81.6)	50 (47.2)
Total (B)	446 (100.0)	446 (100.0)	106 (100.0)
Total (A + B)	542	542	150
**Og4C3 antigen TropBio ELISA**			
**Baseline FTS Positive**			
Negative/ (Seroreversion)	76 (63.3)	88 (73.3)	–
Low Positive	35 (29.2)	24 (20.0)	–
Medium Positive	4 (3.3)	3 (2.5)	–
High Positive	5 (4.2)	5 (4.2)	–
Total	120 (100.0)	120 (100.0)	–

* 1 participant who seroconverted from baseline negative during the first follow-up assessment turned negative at second follow-up.

** The total number of participants who seroreverted included 71 (93.4%) out of the 76 Og4C3 baseline negatives.

Out of the 120 FTS-CFA positive participants at baseline randomly selected for Og4C3 ELISA, 63.3% (n = 76) were negative for Og4C3 antigen ELISA (with only 44 positives; 36.7%), which increased to 73.3% (n = 88) at first follow-up (Table 2). A total of 71 (93.4%) out of the 76 Og4C3 negatives were detected as FTS-CFA negatives only during the first follow-up. No difference was observed for the number of high positive Og4C3 antigen individuals; however, it was observed that four of these individuals who were positive at baseline were the same individuals at follow-up. One initially high positive individual at baseline turned out to be low positive at follow-up, whereas, one medium positive individual moved to high positive. In all, 27.3% (12/44) of the participants initially positive for Og4C3 (35 Low positives, 4 Medium positives and 5 High positives) had become CFA negative. These conversions occurred between 2 to 3 years. Og4C3 TropBio ELISA was not performed for previously FTS-CFA negative participants who were re-tested for a possible seroconversion (Table 2).

### Impact of time (years of follow-up) and additional MDA intake on CFA seroreversion/-conversion

MDA rounds that were taken by participants post-initial screening and follow-up time (since the initial screening) were analysed for their potential impact on antigenemia seroreversion**/-**conversion. Almost 91% (159/175) of the FTS-CFA positive participants had become CFA negative without taking additional MDA during the follow-ups ((86.9%, (152/175) during the first follow-up; and an additional seven (7) out of 22 (31.8%) during the second follow-up)) [Tables 3 and 4]. It was observed that the length of time (period of follow-up) was significantly associated with FTS antigenemia seroreversion in the first follow-up (p < 0.001) but not the second (p = 0.132), with an increasing trend in participants becoming FTS-CFA negative [Tables 3 and 4]. Thus, the rate of seroreversion as assessed by the FTS significantly differed and increased with increasing time, with those followed-up 2–4 years after the initial screening having seroreversion rate of over 70% (Table 3) but this occurrence was not observed during the second follow-up (Table 4). It should be noted that all participants negative at baseline (44, 100.0%) remained negative during the second follow-up assessment (Table 2). Though 27.3% (12 out of the 44 FTS+ participants who were Og4C3 positive at baseline) of the participants assessed for Og4C3 CFA ELISA seroreverted during the first follow-up, the difference was not significant (p = 0.176) and the rate of seroreversion showed no statistical trend (p = 0.557) [Table 5].

**Table 3:** First follow-up–time and additional IVM intake effects on FTS-CFA.

Variable	Groups	Total, N	Positive, N (%)	Negative, N (%)	p-value
**FTS-CFA**					
**Participants Negative by FTS at baseline**		**First Follow-up**			
Years of follow-up after initial screening	2	43	0 (0.0)	43 (100.0)	0.552^a^
	3	15	0 (0.0)	15 (100.0)	
	4	38	1 (2.6)	37 (97.4)	
Additional IVM intake	0 round	58	1 (1.7)	57 (98.3)	1.000^a^
	1 round	7	0 (0.0)	7 (100.0)	
	2 rounds	31	0 (0.0)	31 (100.0)	
Total		96	1 (1.0)	95 (99.0)	
**Participants Positive by FTS at baseline**		**First Follow-up**			
Years of follow-up after initial screening	1	76	30 (39.5)	46 (60.5)	<0.001^a^^,^^b^
	2	82	19 (22.9)	64 (77.1)	
	3	275	33 (12.0)	242 (88.0)	
	4	12	0 (0.0)	12 (100.0)	
Additional IVM intake	0 round	175	23 (13.1)	152 (86.9)	0.030^a^/0.543^b^
	1 round	187	45 (24.1)	142 (75.9)	
	2 rounds	35	8 (22.9)	27 (77.1)	
	3 rounds	49	6 (12.2)	43 (87.8)	
Total		446	82 (18.4)	364 (81.6)	

^a^Fisher’s Exact Test;

^b^Chi-square test for trend (Cochran-Armitage trend test).

**Table 4:** Second follow-up–time and additional IVM intake effects on FTS-CFA.

Participants Positive by FTS at First Follow-up		Second Follow-up			
**FTS-CFA**					
Variable	**Groups**	**Total, N**	**Positive, N (%)**	**Negative, N (%)**	**p-value**
Years of follow-up after initial screening	2	27	19 (70.4)	8 (29.6)	0.132^a^/0.560^b^
	3	18	15 (83.3)	3 (16.7)	
	4	30	22 (73.3)	8 (26.7)	
	5	2	0 (0.0)	2 (100.0)	
Additional IVM intake after initial screening^*^	0 round	22	15 (68.2)	7 (31.8)	0.405^a^/0.586^b^
	1 round	43	34 (79.1)	9 (20.9)	
	2 rounds	8	5 (62.5)	3 (37.5)	
	3 rounds	4	2 (50.0)	2 (50.0)	
Total^**^		77	56 (72.7)	21 (27.3)	

The second follow-up was done to assess a possible change in antigenemia status of participants who still remained FTS-CFA+ during the first follow-up.

* No additional rounds of IVM since the first follow-up assessment were received by any participant during the second follow-up. Second follow-up took place 8-13 months after the first follow-up.

** Total number of FTS-CFA positive participants to have been followed-up for the second follow-up visit was 83 (including the 1 participant who seroconverted from baseline at first follow-up). Six (6) participants still CFA positive at first follow-up were absent for the second follow-up.

All participants negative either at baseline or first follow-up or both (73) were all negative at second follow-up and have been excluded from analysis.

^a^Fisher’s Exact Test;

^b^Chi-square test for trend (Cochran-Armitage trend test).

**Table 5:** First follow-up–time and additional IVM intake effects on Og4C3 CFA.

Participants Positive by FTS at baseline		First Follow-up			
**Og4C3-CFA ELISA**					
Variable	**Groups**	**Total, N**	**Positive, N (%)**	**Negative, N (%)**	**p-value**
Years of follow-up after initial screening	1	50	14 (28.0)	36 (72.0)	0.176^a^/0.557^b^
	2	57	12 (21.1)	45 (78.9)	
	3	13	6 (46.2)	7 (53.8)	
Additional IVM intake	0 round	15	7 (46.7)	8 (53.3)	0.210^a^/0.399^b^
	1 round	92	21 (22.8)	71 (77.2)	
	2 rounds	10	3 (30.0)	7 (70.0)	
	3 rounds	3	1 (33.3)	2 (66.7)	
Total		120	32 (26.7)	88 (73.3)	

^a^Fisher’s Exact Test;

^b^Chi-square test for trend (Cochran-Armitage trend test).

Additional MDA intake by participants followed-up did not change during the two follow-up assessments. Unlike the second follow-up (p = 0.405), the intake of additional MDA during the first follow-up had a significant influence on the outcome of antigenemia seroreversion (FTS-CFA positive to negative) (p = 0.030) [Tables 3 and 4]. However, regarding the Og4C3 CFA ELISA, the proportion of participants who became negative did not differ significantly from those who remained positive (p = 0.210) and showed no trend in seroreversion (p = 0.399) [Table 5].

**Fig 2:**
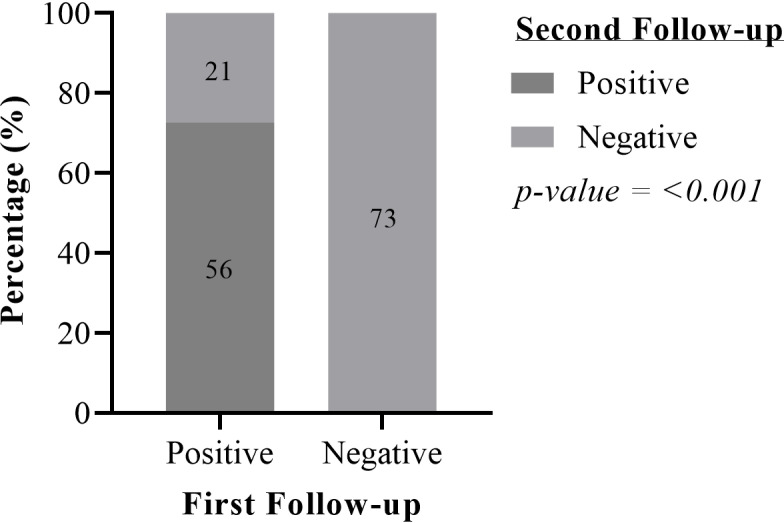
Changes in FTS-CFA status during first and second follow-ups between a period of 8–13 months (McNemar test). One participant who was CFA-negative from baseline became positive at first follow-up but became negative again at the second follow-up.

Regarding antigenemia seroconversion (negative to positive), neither years of follow-up (p = 0.552) nor additional IVM intake (p = 1.000) had a significant influence. A total of 58/96 (60%) of the baseline FTS negative participants assessed for a possible seroconversion did not have additional IVM intake after the initial assessment, with 57 (98.3%) of these maintaining their FTS-CFA negative status at first follow-up. A comparison between the FTS-CFA statuses of participants followed-up twice showed a further significant decline in the number of individuals who seroreverted (p < 0.001). All individuals who were negative during the first follow-up remained negative for the second follow-up (Fig 2). It should be noted that the participant who seroconverted at the first follow-up turned negative again at the second follow-up assessment.

### Changes in antigen levels in FTS-CFA positive participants selected for Og4C3 testing

**Fig 3:**
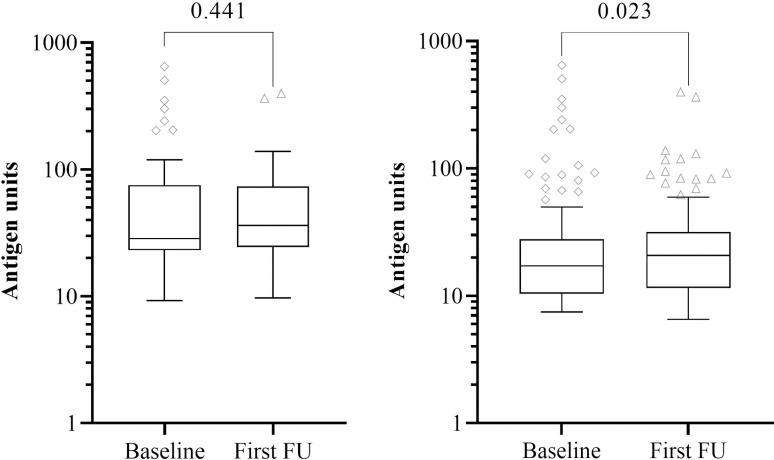
Changes in the Og4C3 antigen levels in baseline FTS-CFA positive participants followed-up (A) individuals positive at both baseline and first follow-up (n = 49) (B) participants positive at baseline but either positive or negative at first follow-up (n = 120). Antigen unit axis expressed in log10 scale. Wilcoxon signed rank test.

Out of the 120 baseline FTS-CFA+ individuals, 71 (59.2%) seroreverted during the first follow-up as tested by the FTS. No statistical difference was observed in antigen levels for individuals positive for FTS-CFA at both baseline and first follow-up (n = 49) [p = 0.441] as tested by TropBio ELISA, although median (interquartile range) values increased from 28.56 (52.09) to 36.10 (48.82) [Fig 3A]. Similarly, the median (interquartile range) antigen levels for the initial screening and first follow-up irrespective of post FTS-CFA status (for the 120 participants) for the Og4C3 antigen were 17.19 (17.45) and 20.74 (20.15) respectively (Fig 3B). This showed a statistically significant increase (p = 0.023) (Fig 3B) and a positive correlation (r = 0.858, p < 0.001) between antigen levels at baseline and follow-up.

### Og4C3 Tropbio ELISA CFA status and levels at baseline and first follow-up

**Fig 4:**
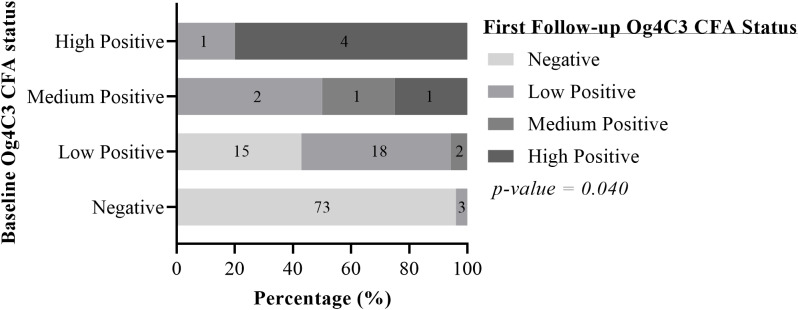
Og4C3 CFA baseline status changes during first follow-up. McNemar-Bowker test.

**Fig 5:**
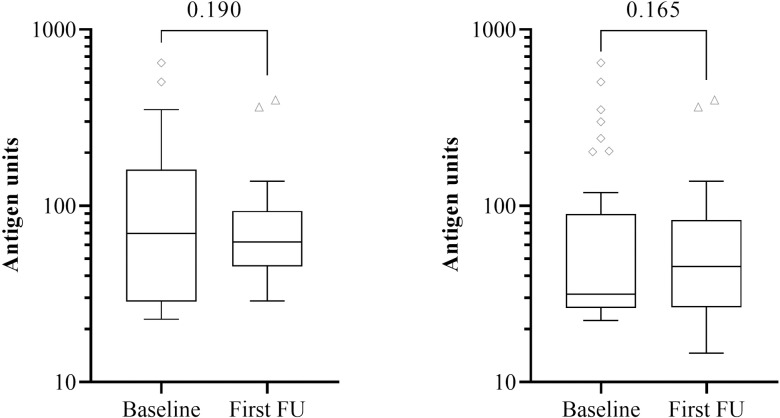
Changes in Og4C3 antigen levels for (A) individuals positive at both baseline and first follow-up (n = 29) (B) participants positive at baseline but either positive or negative at first follow-up (n = 44). Antigen unit axis expressed in log10 scale. Wilcoxon signed rank test.

There was a significant difference between the baseline and first follow-up Og4C3 CFA status of the selected participants (p = 0.040). Almost all participants (n = 73; 96%) who were Og4C3 negative at baseline were also negative at first follow-up, however 42.9% (n = 15) of the participants who were low positives at baseline were negative at the first follow-up. A total of 18 out of 44 (40.9%) previously TropBio Og4C3 ELISA antigen positive participants had comparatively lower antigen levels (15/35 from low positive to negative; 2/4 from medium positive to low positive and 1/5 from high positive to low positive) [Fig 4]. Also, 50% of the participants who tested medium positive for Og4C3 CFA had become low positives. Only 20% of the high positives had their Og4C3 CFA levels lowered (Fig 4). Out of the 120 participants tested for Og4C3 CFA levels, 29 (24.16%) were positive at both baseline and first follow-up. Though the Og4C3 CFA levels (of the 29 participants) had reduced during the first follow-up, there was no statistical difference (p = 0.19) (Fig 5A). Similarly, there was no difference in the Og4C3 CFA levels for participants positive only at baseline but either positive or negative at follow-up (p = 0.165) (Fig 5B).

### Baseline and follow-up CFA and MF characteristics of previous MF positives

Out of six MF positives from the baseline screening [8], three were successfully followed-up with two unavailable (relocated and could not be traced), and one deceased. All three followed-up had become MF negative, with two becoming FTS-CFA negative as well during the first follow-up. However, one individual still remained FTS-CFA positive with a score of 1 both at the first and second follow-ups (Table 6). The two participants who had seroreverted and thus had become both CFA and MF negative, were followed-up 3 and 4 years after the initial screening. The participant that remained CFA+ but MF- was followed-up 2 and 3 years after the first screening (for first and second follow-ups respectively) and all three participants had each taken IVM twice after their initial screening (Table 6). Available antigen units from the Og4C3 TropBIO ELISA testing for these microfilaremia participants ranged between 19.652 AU to 64.410 AU.

**Table 6:** Baseline and first follow-up CFA and MF characteristics of previous MF positives.

PID	Age	Sex	Baseline FTS-CFA status	Baseline MF assessment/count		First Follow-up FTS-CFA status (score)	First Follow-up MF status (Sedgewick and nucleopore
				Sedgewick (Mf/200µL of blood)	Nucleopore (Mf/mL of blood)		
A^a^	32	M	Positive	0	1	Negative (0)	0
B	48	F	Positive	1	1	Unavailable	—
C^b^	55	M	Positive	5	15	Negative (0)	0
D	45	F	Positive	11	85	Unavailable	—
E	58	F	Positive	0	3	Deceased	—
F^c^	42	M	Positive	11	54	Positive (1)	0

^a^Followed-up after 3 years;

^b^Followed-up after 4 years;

^c^Followed-up after 2 years. Unavailable participants had relocated and could not be traced for both follow-ups. Participa F was FTS-CFA positive with a score of 1 at both follow-ups. Participant A had an antigen unit of 19.652 AU; B had 22.406 AU; C had 43.124 AU; D had 64.410 AU; E had 42.942 AU and F had 31.333 AU.

## Discussion

Most LF sentinel sites that have been undertaking regular annual or biannual MDA are characterized by typically low MF prevalence but relatively high antigen prevalence, despite several years of preventive chemotherapy [8,14]. This study examined the impact and influence of time and additional MDA intake on LF antigenemia seroreversion (change in status from antigen positive to -negative) and seroconversion (change in status from antigen-negative to -positive) in 542 individuals who had previously tested positive (N = 446) and negative (N = 96) to the Alere FTS test. In our previous study, the CFA prevalence as assessed using the Alere FTS was 19.6% and 12.8% (KNEM and Nabdam, respectively) after >15 rounds of MDA, and the projected Og4C3 antigen prevalence was 3.8% for the two districts despite a comparatively very low MF positives (0.14%)—thus failing the recommended < 2% CFA threshold required to pass pre-TAS [8]. Overall, from the two follow-up visits in this present study; 159 out of the 175 (90.9%) participants who were previously antigen (FTS-CFA) positive and did not receive additional ivermectin/Albendazole and also 226 out of the 271 (83.4%) with varying additional MDA treatments seroreverted.

During the first follow-up study, a significant proportion (81.6%) of the study participants who were CFA positives using the WHO recommended FTS had become antigen (CFA) negative (seroreverted). A further 27.2% (21/77) of the remaining FTS-CFA positives had also seroreverted during the second follow-up which was undertaken 8–13 months after the first follow-up. Also, 27.2% (12/44) of the participants who were previously Og4C3 TropBio ELISA-antigen positives had entirely become negative within the same period. Additionally, almost 41% (18/44) of the participants had their Og4C3 TropBio ELISA antigen levels reduced (Fig 4). Also, a trend of reduction, albeit not significant (p = 0.19), in Og4C3 CFA levels of participants who were FTS positive at both the initial screening and follow-up was observed in this study (Fig 5A). Regarding the incidence of MF, all 3 previously positive participants followed-up had become amicrofilaremic, with 2 also becoming entirely FTS-antigenemia negative. A higher rate of FTS/Og4C3 TropBio ELISA antigen seroreversion was observed for the KNEM district in comparison to the Nabdam district. This could be attributed to the impact of several more rounds of MDA in KNEM compared to Nabdam, as KNEM was one of the first districts to have started MDA in Ghana, in the year 2000 [5].

Consequently, the observed low occurrence of the disease’s parasitological indices (CFA and MF), in these two LF hotspot districts suggests a general downward trend in endemicity of LF infection in the study districts in the course of time, after many years of preventive chemotherapy with ivermectin and albendazole [8]. Though time (years of follow-up) [p < 0.001] and additional MDA rounds taken (p = 0.030) both had significant impacts on LF FTS-antigenemia seroreversion, the influence of follow-up years (time) was much more compelling―with additional IVM intake having minimal influence. On the contrary, time (years of follow-up) and additional MDA intake did not have a significant influence on the seroreversion and reduction/change of antigenemia levels/status in participants tested by the Og4C3 TropBio ELISA in this follow-up study. This might be due to the fact that though there were seroreversion (27.2%; 12/44) and reduction (41%; 18/44) in Og4C3 CFA-antigen levels in participants during the follow-up, majority [63.3% (76/120)] of the FTS-CFA positive participants randomly selected for Og4C3 CFA assessment were baseline Og4C3 negatives. This shows that the Og4C3 TropBio ELISA is robust to changes in endemicity status of mapped LF districts as determined by the FTS, with Og4C3 TropBio ELISA having high specificity for active and current LF infections as compared to the FTS [7,11,15].

All FTS-CFA positive participants (N = 14) who were followed-up 4 years (N = 12 for the first follow-up) and 5 years (N = 2 for the second follow-up) afterwards had seroreverted, whereas 60.5% of participants followed-up a year after initial screening in the first follow-up had also seroreverted. The rate of antigenemia seroreversion increased as the years of follow-up also increased during the first follow-up. Moreover, there was a reduction in the antigenemia levels of participants who were still FTS positive during the first follow-up in the current study. It must however be noted that approximately 91% (in both follow-ups) of the previously FTS-CFA positive participants who did not have additional MDA after the initial screening had also seroreverted. Moreso, there was no antigenemia seroconversion in over 98% of the participants who were previously FTS-CFA negative despite no additional MDA intake. Also, none of the previously/baseline FTS-CFA negative participants had become antigen positive during the second and final follow-up—suggesting the absence of infection/or re-introduction among the study cohort over the years. It must be said that as a limitation to the study, the number of FTS negative participants who consented to participate in the study lacked statistical power as compared to the number of FTS positive participants recruited. However, the results of this study suggest that in the natural course of time, many (or all) previously FTS-CFA positive individuals, who have participated in past MDA programmes would eventually serorevert (become CFA negative) without an additional MDA. This proposition is strongly supported by the fact that a further 27.3% (n = 21/77) of the remaining FTS-CFA positives during the second follow-up (conducted 8–13 months after the first follow-up) had also seroreverted without an additional MDA intake (since no MDA treatment took place between the first and second follow-ups). However, the subsequent MDA intake (especially by those with ≥5 rounds, the number recommended by WHO) by some study participants before the first follow-up might have also played a role in antigenemia seroreversion during the second follow-up, which might also support the frequently reported effectiveness of the current MDA programmes [2,5].

The unresolved concern about the “accuracy” of the Alere FTS diagnostic test in detecting active bancroftian infection [16] remains a dilemma for the LF elimination programme. A plausible explanation for the study participants’ initial CFA positivity detected by the FTS in our previous screening [8], might be as a result of detection of persistent residual circulating filarial antigens from dead adult worms, even when at lower concentrations, as have also been reported elsewhere [11,15]. The FTS diagnostic tool, as other studies have already revealed, is more sensitive [8,17] primarily due to its detection of residual target antigens which are cleared slowly from general blood circulation as reported by others [11,12]. Thus, the FTS is more likely to identify individuals with low CFA levels than the Og4C3. It is therefore uncertain if the high FTS-CFA prevalence reported during the initial screening [8], after several MDA rounds in these LF-endemic districts, truly reflected an ongoing LF infection transmission that warrants additional MDA programme interventions, or that the FTS only detected remnants of antigens which were still in general blood circulation due to their slow clearance [11,12]. However, the latter seems more likely per the results of this study as almost 87% (152/175) of study participants who did not have additional MDA drugs taken had seroreverted during the first follow-up (with 81.6% of the overall participants seroreverting), with an additional 27.3% (21/77) also seroreverting in the second follow-up without an additional MDA intake. This assertion is further strengthened by the fact that an estimated 63% (76/120) of the FTS antigen positives assessed by the Og4C3 TropBio ELISA at the initial screening were Og4C3-CFA negatives (Table 2 and Fig 4). The FTS however, detected majority (71 out of 76; 93.4%) of these as CFA negatives only in this current follow-up assessment―many of which had not taken additional MDA within the follow-up period. This observation supports the opinion among many control programme implementers that the FTS may not be too “reliable” a diagnostic tool for measuring MDA programme success and undertaking pre-TAS in sentinel sites like KNEM, which had higher baseline prevalence, prior to MDA commencement in 2000 [16].

Direct comparison of the FTS and Og4C3 ELISA results in this current follow-up study shows a stronger level of agreement between the two tests (after a lot of FTS antigenemia seroreversions even without additional MDA), indicating a similar prevalence-estimate for the two tests in the current study in comparison to that of the initial screening — where the Og4C3 ELISA showed positive results in only about one-fifth of the FTS-CFA positive individuals [8]. This finding suggests that the Og4C3 TropBio ELISA may be better suited for the early detection of CFA positive to negative seroreversion compared to the FTS.

To achieve LF elimination, WHO through the GPELF first recommends pre-TAS in sentinel and spot-check sites after more than 5 effective MDA rounds covering over 65% of the total endemic population [2,18]. If pre-TAS shows that prevalence in each site has been lowered to less than 1% MF or less than 2% antigenemia, the implementation unit conducts a Transmission Assessment Survey (TAS) to determine whether MDA can be stopped [16,18–20]. However, failure to pass the pre-TAS, which is the initial assessment, means that further rounds of MDA are required [16,18–20]. A recent study indicated an increased risk of pre-TAS failure when the FTS diagnostic kit is used to assess treatment impact on antigenemia levels among adults [16]. Thus, it may become increasingly difficult for sentinel and spot-check-sites with less than 1% MF prevalence such as Nabdam and KNEM, to pass pre-TAS and ultimately TAS, based on the CFA index if the FTS diagnostic kit is continually used in transmission assessment. This has necessitated the need for a more rigorous evaluation of the diagnostic performance of the FTS in monitoring active bancroftian infection and treatment success for policy consideration in sentinel and spot-check sites.

The need for high-quality diagnostic approaches to accurately assess MDA programme status in LF-endemic areas was recognized by the Diagnostic Technical Advisory Group of the WHO [21]. Proposals for serial testing using multiple diagnostic tools have been suggested to compensate for the reported specificity flaw presented by the FTS in low prevalent settings such as KNEM and Nabdam which have undergone several MDA rounds [22]. The high specificity of the Og4C3 TropBio ELISA shown in this study highlights the significant value of this test to confirm FTS-CFA positive individuals who are truly infected and thus present a true burden of the infection. However, the Og4C3 TropBio ELISA approach is associated with increased costs of testing, sample collection and transportation, and above all not field-friendly [22]. Despite these difficulties, the Og4C3 TropBio ELISA is an important tool for post-MDA surveillance in low MF settings, because it limits the number of false positives detected by the FTS that could trigger unwarranted additional treatment rounds and follow-ups that may not drastically reduce antigenemia but rather burden the district with valuable financial and human resources required for drug distribution, supervision and monitoring to implement these further unwarranted MDA rounds. Also, Og4C3 TropBio ELISA provides the quantitative measures of CFA used as a proxy to monitor the progress of antifilarial treatment. Instead, a ‘Test-and-Treat’ approach could be explored, where true CFA positive individuals are identified by confirming FTS-CFA positives with the Og4C3 TropBio ELISA in sentinel sites with less than 1% MF and treating these individuals alternatively with a potent macrofilaricidal drug such as doxycycline, as was done in OEPA [23], or with triple therapy (ivermectin plus diethylcarbamazine plus albendazole) [11]―which would cause the non-fecund adult worms (if indeed they are present) to degenerate early, yielding a rapid decline in CFA levels and a subsequent drastic reduction in antigenemia thereafter.

Alternatively, as it was suggested and recommended in our previous report [8] and also elsewhere [14], the long-standing less than 2% CFA threshold needed to pass pre-TAS may be too stringent and thus may need a revision in this end-game period. This has become necessary especially after more sensitive diagnostic kits such as the Alere FTS, which has the propensity to detect the slow-clearing residual circulating filarial antigens from dead worms in the blood [11,12], have been introduced for post-treatment surveillance and measurement of MDA programme success [2,18–20]. This consequently has made it increasingly difficult for most sentinel sites with less than 1% MF prevalence, such as KNEM and Nabdam, to pass pre-TAS based on the FTS-CFA index. Thus, the revision of the less than 2% CFA threshold (to >2% but <5%) by WHO may help avoid further treatment rounds in these sites which might not be needed [16]. Also. finding a threshold concentration (antigen unit/level) at which the FTS will likely produce false positive results in this end-game scenarios in endemic foci with several MDA rounds would help guide decision making by the LF control and elimination programme.

Xenomonitoring (PCR-based detection of MF DNA in vector mosquitoes) could also be conducted in these areas to ascertain the absence of ongoing LF transmission.

Generally, the rate of CFA seroreversion should be directly proportional to the rate of CFA antigenemia level reduction following successful treatment interventions [24]. CFA seroreversion was seen for both FTS and TropBio ELISA tests, albeit no statistically significant reduction in overall antigenemia levels of individuals was observed during follow-up in the study cohort as assessed by TropBio ELISA (likely owing to the fact that majority of the FTS+ individuals selected for the Og4C3 TropBio ELISA assessment were Og4C3 CFA negatives at baseline). The female-reproducing adult worms are the main producers of CFA [24]. Hence, the lack of effect of MDA, which is primarily microfilaricidal, on antigenemia levels suggests that the adult worms (if indeed present) in the FTS/TropBio ELISA positive individuals were non-fecund, and thus would not cause disease transmission. This assertion is further strengthened by the lack of MF in the study cohort. Regardless, the impact and influence of years of follow-up (time) on antigen seroreversion was significant and therefore we encourage re-assessment studies in sentinel and spot-checks to be done at least 4–5 years after these sites have achieved MF prevalence of less than 1% after several years of MDA with ivermectin and albendazole as the remnants of the dead worm antigens should have been depleted by then.

## Conclusion

The findings of this study suggest that many (or all) previously FTS-CFA positive individuals, who have participated in past MDA programmes will eventually serorevert over the course of time. Furthermore, the study demonstrated that in comparison to the FTS, the Og4C3 TropBio ELISA may be more accurate and well-suited for the early detection of CFA positive to negative seroreversion, due to the unresolved concern regarding the “specificity” of the FTS in detecting active bancroftian infection—and thus may prevent further unwarranted MDA/treatment rounds. Also, the recommended less than 2% CFA positivity threshold required to pass pre-TAS may be too stringent in remaining endemic foci, and may need revisions (>2 but <5) in end-game scenarios, particularly in light of the introduction of more sensitive diagnostic tools like the FTS that has the propensity to detect slowly clearing residual CFA in the blood as evidenced in this study. Regardless, the impact and influence of time on CFA seroreversion was highly significant in comparison to additional ivermectin/Albendazole intake. Thus, we recommend re-assessment studies in sentinel sites to be conducted ≥4–5 years after these sites have achieved MF prevalence of <1% as the residual CFA should have been cleared from the blood.

## Supporting information

S1 Table (A) Demographic characteristics of participants who had never received MDA treatment; (B) Demographic characteristics of participants with 1-4 MDA treatment rounds. (DOCX)
