# Supplementary material for: Changes in circulating filarial antigen status in previously positive individuals: Lessons for treatment monitoring and pre-transmission assessment surveys
Source: PLoS Negl Trop Dis. 2025 Feb 4;19(2):e0012802. doi: 10.1371/journal.pntd.0012802 (PMC12129320; doi:10.1371/journal.pntd.0012802)
Supplement: S1 Table — (DOCX) [file pntd.0012802.s001.docx]

**Table A: Demographic data of participants who had never received MDA treatment.**

| Variable | Category | Never Taken IVM/ALB  N (%) | Baseline FTS-CFA Positivity  N (%) | First Follow-up FTS-CFA Positivity  N (%) |
| --- | --- | --- | --- | --- |
| Gender | Male | 15 (55.6) | 13 (86.7) | 3 (20.0) |
|  | Female | 12 (44.4) | 11(91.7) | 0 (0.0) |
| Age groups (years) | 14-30 | 4 (14.8) | 3 (75.0) | 1 (25.0) |
|  | 31-40 | 9 (33.3) | 9 (100.0) | 1 (11.1) |
|  | 41-50 | 11 (40.7) | 9 (81.8) | 1 (9.1) |
|  | ≥ 51 | 3 (11.1) | 3 (100.0) | 0 (0.0) |
| Total |  | **27 (100.0)** | **24 (88.9)** | **3 (11.1)** |

**Table B: Demographic characteristics of participants with 1-4 MDA treatment rounds.**

| Variable | Category | 1-4 rounds IVM/ALB  N (%) | Baseline FTS-CFA Positive  N (%) | First Follow-up FTS-CFA Positive  N (%) |
| --- | --- | --- | --- | --- |
| Gender | Male | 107 (49.8) | 86 (80.4) | 17 (15.9) |
|  | Female | 108 (50.2) | 78 (72.2) | 13 (12.0) |
| Age groups (years) | 14-30 | 58 (27.0) | 38 (65.5) | 3 (5.2) |
|  | 31-40 | 64 (29.8) | 51 (79.7) | 11 (17.2) |
|  | 41-50 | 62 (28.8) | 52 (83.9) | 10 (16.1) |
|  | ≥ 51 | 31 (14.4) | 23 (74.2) | 6 (19.4) |
| Total |  | **215 (100.0)** | **164 (76.3)** | **30 (14.0)** |
